# Health Scares: Tracing Their Nature, Growth and Spread

**DOI:** 10.32872/cpe.12209

**Published:** 2023-12-22

**Authors:** Kate MacKrill, Michael Witthöft, Simon Wessely, Keith J. Petrie

**Affiliations:** 1 Department of Psychological Medicine, Faculty of Medical and Health Sciences, University of Auckland, Auckland, New Zealand; 2 Department of Clinical Psychology, Psychotherapy and Experimental Psychopathology, Johannes Gutenberg University of Mainz, Mainz, Germany; 3 Department of Psychological Medicine, Institute of Psychiatry, Psychology & Neuroscience, King’s College London, London, United Kingdom; Philipps-University of Marburg, Marburg, Germany

**Keywords:** health scares, media, environmental incidents, technology, nocebo effect

## Abstract

**Background:**

Health scares are highly publicised threats to health that increase public concern and protective behaviours but are later shown to be unfounded. Although health scares have become more common in recent times, they have received very little research attention. This is despite the fact that health scares often have negative outcomes for individuals and community by affecting health behaviours and causing high levels of often unnecessary anxiety.

**Method:**

In this paper we undertook a review and analysis of the major types of health scares as well as the background factors associated with health scares and their spread.

**Results:**

We found most health scares fell into seven main categories; environmental contaminants, food, malicious incidents, medical treatments, public health interventions, radiation from technology and exotic diseases. For most health scares there are important background factors and incident characteristics that affect how they develop. Background factors include conspiracy theories, trust in governmental agencies, anxiety, modern health worries and wariness of chemicals. Incident characteristic include being newly developed, not understood or unseen, man-made rather than natural and whether the incident is out of personal control. We also identified the aspects of traditional and social media that exacerbate the rapid spread of health scares.

**Conclusion:**

More research is needed to identify the characteristics of media stories that intensify the levels of public concern. Guidelines around the media’s reporting of health incidents and potential health threats may be necessary in order to reduce levels of public anxiety and the negative public health impact of health scares.

## Highlights

- Most health scares can be categorised into one of seven types.
- Underlying environment and social factors influence the development of health scares.
- Traditional and social media could reduce the negative public health impact of health scares.

We live in the age of health scares, defined as a highly publicised threat (or perceived threat) to health that causes increases in public concern, avoidance or protective behaviour but is substantially disproportionate to the risk involved (MacKrill, 2021). News stories frequently appear in the media that raise concern about common household products, food or medication. While the respective health risk appears frighteningly large initially, it turns out to be comparatively low or unfounded in retrospect (Hooker, 2010). The early stage of a health scare is characterised by an increase in concerns and public anxiety which is followed by the gradual reduction in the frequency and tone of coverage, until the event is no longer newsworthy. Consequently, it is the response of the public and the media that elevates a health incident into a scare (Whitworth et al., 2017).

Many health scares involve the unexpected dangers of modern or new technology, such as 5G or Wi-Fi. Others, such as in modern food production, are concerned with chemical additives, processing or colourings. Most countries have experienced some form of scare over artificial sweeteners, the overuse of antibiotics in food, and genetically modified ingredients. Modern medicine has also been implicated, with drugs and other medical treatments always being a prominent source of public concern. Anxieties about vaccination are as old as the intervention itself but have recently gained more visibility with the COVID-19 pandemic.

Understanding the nature of health scares has become more important with increasing recognition and numerous examples of the public health consequences they can have. For example, the COVID-19 crisis has provided many unfortunate illustrations of how unfounded health scares about the virus and its control can cause negative outcomes for individuals and communities. These included the belief that COVID-19 was spread by 5G towers, which lead to a number of towers being damaged (Ahmed et al., 2020). A related scare was that COVID-19 vaccines alter people’s DNA and that the vaccine was developed to control individuals by placing a microchip inside them for easy tracking (Sanders, 2020). This has contributed to a greater hesitancy for some individuals to be vaccinated and consequently affected uptake and community immunity.

Health scares can be rapidly transmitted on informal social networks spreading further anxiety and negative expectations in a community (Southwell et al., 2019). Research suggests that individuals who hold strong conspiracy beliefs also are more likely to believe that some of the same factors that are commonly associated with health scares also cause cancer, such as eating genetically modified food and microwave ovens (Paytubi et al., 2022). The transmission of these beliefs can establish negative expectations that may subsequently produce a nocebo effect when the individual has been exposed to the focus of concern (Crichton, Dodd, et al., 2014). A nocebo effect is defined as adverse effects that are caused by negative expectations rather than any physical effects from exposure to an object of concern (Petrie & Rief, 2019).

In this paper we start by providing a taxonomy of common health scares, followed by an analysis of the background factors and circumstances associated with their development, as well factors involved in their spread. We end with a discussion of areas for future research on health scares.

## Taxonomy of Health Scares

Research on health scares identifies particular health interventions, consumer goods or features of modern life that are often the subject of unexplained adverse reactions or unfounded concerns. From our analysis of this literature, we found that health scares fell into seven main categories; environmental contaminants, food, malicious incidents, medical treatments, public health interventions, radiation from technology and exotic diseases (Table 1). Consistent across many health scares is the fact that they can arise from a legitimate concern but what distinguishes health scares is the disconnect between the level of perceived and actual risk.

**Table 1:** Taxonomy of Common Health Scare Areas With Examples of Health Concerns and Evidence for the Scare

Health Scare	Reference Examples
Environmental contaminants	
Building ventilation/sick building	Kinman & Griffin, 2008; Mendelson et al., 2000; Ooi & Goh, 1997
Water scares	Banner, 2018; David & Wessely, 1995; Petrie & Wessely, 2004; Roy et al., 2023
Wind turbine infrasound	Chapman et al., 2013; Crichton, Chapman, et al., 2014
Exotic diseases	
Swine flu	Klemm et al., 2016
SARS	Hooker, 2008; Tausczik et al., 2012
Food scares	
Genetically modified food	Frewer et al., 2002; Shaw, 2002
Food contamination	Jacob et al., 2011
Additives	Bearth et al., 2014; Haen, 2014
Malicious incidents	
Anthrax	Leask et al., 2003; Wills et al., 2008
Deliberate chemical or radiation poisoning	Rubin et al., 2007; Rubin & Dickmann, 2010; Rubin et al., 2020
Medical treatments	
Amalgam fillings	Dodes, 2001; Flanders, 1992; Molin, 1992
Generic drugs and medicine reformulation	Boone et al., 2018; Faasse et al., 2009; Faasse et al., 2012; MacKrill et al., 2019
Hormone replacement therapy	Bluming & Tavris, 2009; Haas et al., 2007
Public health interventions	
Vaccination programmes	Burgess et al., 2006; MacKrill, 2023; Petts & Niemeyer, 2004
Water fluoridation	Armfield, 2007; Carstairs & Elder, 2008; Howat et al., 2015
Radiation from technology	
Electromagnetic fields	Rubin et al., 2010
Wi-Fi	Bräscher et al., 2017; Bräscher et al., 2020; Witthöft & Rubin, 2013
5G	Foster, 2019
Mobile phones and towers	Burgess, 2004; Drake, 2006; INTERPHONE Study Group, 2010

*Note.* Adapted from MacKrill (2021).

The first category of environmental contaminants involves instances where the public believe they have been exposed to a noxious substance in the environment, such as infrasound from wind turbines or chemicals in drinking water. These exposures are unlikely to have a physical effect on health but people report symptoms due to the perception of harm (Crichton, Chapman, et al., 2014; David & Wessely, 1995).

Health scares relating to exotic diseases occur when it is retrospectively determined that the catastrophic outcomes initially predicted when the disease first appeared did not occur. For example, the World Health Organisation (WHO) warned in 2004 that the bird flu virus could kill millions of people (Bird, 2005), however, to date there has been 457 deaths globally (WHO, 2023). Potentially this category represents an important dilemma that robust and effective prevention strategies that are used to contain an outbreak or virus could potentially increase anxiety and contribute to the creation of a health scare, as the public may see preventative measures as a sign of a severe and imminent health threat.

Food scares involve concerns that additives in food or genetic modification cause health problems. Scares may also occur when there is a confirmed contamination case but the public responds by avoiding foods unrelated to the incident (Whitworth et al., 2017). On a broader level various foods containing gluten, dairy, flavourings, lactose, and various additives, such as sulphites can also cause avoidance and anxiety from time to time (Haen, 2014; Vernia et al., 2010).

A further category is comprised of scares involving malicious incidents. This category is characterised by targeted attacks on individuals that involve methods like radiation that could potentially impact the wider community and cause significant public anxiety, even if the actual risk of harm to the wider public is low. After the poisoning of the former Russian secret service agent Alexander Litvininko in London in 2006, a survey found 12% of Londoners believed their own health was at risk due to the Polonium poisoning (Rubin et al., 2007). More recently following the deliberate poisoning of an ex-Russian intelligence officer and his daughter with Novichok in Salisbury, 19% of a sample of Salisbury locals reported avoiding the city despite it being a targeted rather than random event (Rubin et al., 2020). A large number of people with anxiety and distress-induced symptoms seek medical care following a terrorist attack or other malicious incident (Engel et al., 2007).

Two categories relate to medical interventions, namely concerns about medical treatments, and worries about public health interventions. Scares involving medical treatments typically involve existing patient groups, where the treatment is subsequently revealed to have unexpected side effects or undergoes changes in ingredient formulation or appearance, which can elicit a nocebo response due to negative expectations (Faasse et al., 2009; Faasse et al., 2016). In regards to scares relating to public health interventions, these occur in non-patient groups receiving a medical treatment, such as in the case of large vaccination campaigns. This can foster worries and reluctance, as people experience no visible benefit, such as symptom reduction, and are instead exposing themselves or vulnerable others (such as in childhood vaccinations) to potential adverse reactions or the risk of unforeseen negative effects (Martin & Petrie, 2017; Petts & Niemeyer, 2004).

Scares involving radiation from technology centre on the perceived harm of invisible electromagnetic fields, such as those from mobile phones, Wi-Fi or 5G, which do not have a physical effect no health (Rubin et al., 2010). A previous study has shown that when highly anxious participants are shown a television documentary about the possible health effects of Wi-Fi they are more likely to report symptoms after exposure to a sham Wi-Fi signal and to decide they were sensitive to electromagnetic fields (Witthöft & Rubin, 2013).

The health scare taxonomy differentiates the primary areas of concern in seven main categories. Table 1 provides an illustration of the common health scares in each category, references to specific examples of health concerns, and evidence for the scare. However, we recognise this is to some extent an arbitrary categorisation. The categories could easily be divided further, which has been done for food scares by Page and colleagues (2006) and Whitworth et al. (2017) for environmental contaminations. It is important to note that health scares are typically wider and affect a greater number of people than incidents of mass psychogenic illness (MPI), which occur after a discrete event involving a closed community, such as a school or office building.

## Background Factors

Health scares do not occur in a vacuum and are instead produced through the environment and social context that effects their development and spread. We term these the background factors, which shape an individual’s interpretation of threat and expectations about how their health may be affected. In this section we discuss a number of factors in the contemporary social environment as well as individual factors that influence the impact and spread of health scares, specifically: conspiracy theories, trust in governmental agencies, anxiety, modern health worries, and a wariness of chemicals.

### Conspiracy Theories

While it is clear that conspiracy theories have been with us for as long as there have been theories, there is evidence that they increase during periods of uncertainty and threat as has been the experience for many during the COVID-19 pandemic (van Prooijen & Douglas, 2017). Often conspiracy theories, which ascribe events to malevolent people or powers, provide a ready-made explanation of events that may be threatening or anxiety provoking, providing a simplistic, albeit wrong, explanation of complex events (Aaronovitch, 2010).

A conspiracy mentality, or the tendency to believe conspiracy theories, has important health consequences and is likely to influence the spread of health scares. Oliver and Wood (2014) found medical conspiracy theories, such as the FDA is deliberately suppressing evidence about natural cures of cancer because of pressure from drug companies, to be common in the US population. Conspiracy beliefs are associated with a wide range of health behaviours, such as preferences for organic food and avoidance of mainstream medicine (Oliver & Wood, 2014). There is evidence that the acceptance of conspiracy theories is associated with a shunning of vaccination (Jolley & Douglas, 2014) and lower adoption of recommended preventative actions against COVID-19 such as wearing a face mask (Romer & Jamieson, 2020).

### Trust in Governmental Agencies

A similar influential factor is the degree to which people trust governmental agencies. Suspicion and distrust of government institutions makes reassurance from official channels less effective following a health scare (Uscinski et al., 2016). Distrust in the healthcare system is associated with a greater tendency to believe health misinformation (Scherer et al., 2021). In the Salisbury Novichok incident, lower trust in governmental agencies was associated with greater anxiety, perceived risk to self, and an increased likelihood of avoiding Salisbury (Rubin et al., 2020). Trust can also affect side effect reporting. In an experimental study, lower trust in pharmaceutical regulatory agencies was associated with a greater number of side effects being attributed to a placebo tablet (Webster et al., 2018). In a medicine brand change, lower trust in pharmaceutical agencies was associated with a lower belief in the efficacy of a new generic medicine (MacKrill & Petrie, 2018).

### Anxiety

Anxiety is, by definition, associated with health scares. The publicization of health threats increases the general public’s anxiety but existing trait anxiety can be an important background to factor in the development of health scares. Anxiety has a close relationship with the tendency to notice physical symptoms and to interpret them more negatively (Barsky et al., 2002; Watson & Pennebaker, 1989). It is this misattribution process that is key in health scares and research suggests individuals higher in anxiety not only experience a greater number of symptoms but there is a greater tendency to misattribute these to the effects of any given health scare (Faasse et al., 2009; Petrie et al., 2004; Witthöft & Rubin, 2013).

### Modern Health Worries

Concerns related specifically to modernity or new technology have also been identified as a risk factor for health scares generally (Petrie et al., 2001). Modern health worries are surprisingly prevalent with a large proportion of people acknowledging concerns about the safety of food or the health effects of chemicals in household products. A German study found that 94% of people report some concerns about the effect of modernity on health and that this was associated with greater symptom reporting (Rief et al., 2012). Other studies have found higher levels of modern health worries to be associated with a greater use of organic food and alternative medicine (Devcich et al., 2007; Furnham, 2007). The influence of modern health worries in a particular health scare was examined in a prospective study looking at the health effects of an aerial pesticide spray programme to control an invasive moth species in New Zealand. Individuals with higher levels of modern health worries were found to attribute more symptoms to the spray programme and to also believe the spray caused more health problems for themselves, their children and pets compared to those with lower levels of modern health worries (Petrie et al., 2005).

### Wariness of Chemicals

A related factor is an increase in the fear of chemicals or the association of chemicals with cancer, death and toxicity (Siegrist & Bearth, 2019). This has been called “toxicohistrionics” (Banner, 2018) and is often associated with the belief that modern manufacturing produces products that have dangerous levels of chemical substances that are hazardous to health (Saleh et al., 2019) and may be particularly associated with water and food-related health scares. Negative attitudes towards chemicals are associated with a greater preference for natural foods (Dickson-Spillmann et al., 2011). People with high levels of concerns about chemical substances that are present in food or the environment often do not consider the importance of dose (the dose makes the poison) or that the distinction between synthetic and natural chemicals is irrelevant when assessing chemical risk in food (Paarlberg, 2021) or water (Roy et al., 2023). It seems that this concern is increasing while the risk of such exposures has decreased over time (Entine, 2011).

## Incident Characteristics

Background factors are only one part of the foundation required to develop health scares. Health-related worries on their own will not manifest symptoms, rather a threatening event is also required to influence bodily awareness and the misattribution of symptoms to a particular category of scare. For instance, worry about power lines did not influence symptom reporting for people who did not live next to high voltage transmission lines, whereas for those living in these areas, the most worried respondents were more likely to report health problems (McMahan & Meyer, 1995). It seems logical that background factors alone can’t create a health scare and that a threatening event is also necessary. However, not all health interventions or environmental events will be perceived as threatening. Incidents that often develop into larger health scares have certain characteristics that instil worry. These characteristics include: being newly developed, not well understood or unseen threats, natural versus man-made, and out of personal control (MacKrill, 2021).

### Newly Developed

A frequent unifying factor of many common examples of health scares is that the event or medical intervention is modern or newly developed. Through history it is evident that health scares often follow the advent of a new form of technology. When the bicycle was created in the 1880s it was believed that the riding position would cause hernias and curvature of the spine and that women in particular could become possessed by ‘cyclemania’ (Whorton, 1978). As the novelty of the technology begins to decline so does concern; it is now accepted that cycling conveys many health benefits. Anxiety and concerns surrounding modernity still exist but the focus has shifted towards the latest technological advancement such as 5G (Elwood & Wood, 2019). It should be noted that this can also include changes to existing familiar interventions, such as medications, that take on a new form or colour (Faasse et al., 2009).

### Not Understood and Unseen

It is often the case with newly developed technology that the underlying science is not be well understood by lay people. The general public may believe that the safety of the intervention has not been proven and unidentified negative effects might still occur. An example here is the new mRNA COVID-19 vaccines that use new technology to produce an immune response. The public’s confusion and concern about the potential unknown effects of these interventions can be fuelled by the perception of ‘unseen’ harms. For example, these interventions are often described as an invisible danger and people can be concerned that they are being unknowingly exposed to a perceived health threat (e.g., Owens & Feldman, 2004; Reekie, 2017).

This has also been the case for health scares about electromagnetic fields (EMFs), such as those from Wi-Fi, mobile phones and microwave ovens. Public discussion about the health effects of EMFs has focused on the radiation emitted, with claims that mobile phones or Wi-Fi can cause cancer (Swerdlow et al., 2011). Despite the widespread use of phones there has actually been a decrease in the diagnosis of brain and other nervous system cancers over the last 15 years in the United States (United States Food and Drug Administration, 2020). Confusion can be further exacerbated through factually correct albeit unclear information like the World Health Organisation (WHO) classifying mobile phones as “possibly carcinogenic” (WHO, 2014). While this sounds alarming to the general public, there are other normal, everyday things, like pickled vegetables and carpentry, that also share this classification.

### Man-Made Versus Natural

In a similar vein is the differing perception of harm from natural versus man-made interventions. There is a common misconception that synthetic chemicals, at any concentration, are harmful (Entine, 2011). Chemicals of natural origin are perceived to be healthier and safer than synthetic chemicals, since the latter involve human intervention (Saleh et al., 2019). Even though a medicine’s efficacy and safety may be clinically proven, patients can be fearful of putting ‘unnatural chemicals’ into their bodies and instead turn to untested ‘natural’ remedies (Petrie & Wessely, 2002).

### Low Personal Control

If a situation is perceived to be out of an individual’s control then this can also promote health scares. The perception of threat can be high when an incident is uncontrollable (Slovic, 1987). A feeling of lacking personal agency can occur through the government acting on behalf of the public. This is the case with water fluoridation, which despite the overwhelming evidence that fluoride is safe and effective at reducing tooth decay, is viewed as a violation of people’s rights not to be subjected to compulsory medication (Reekie, 2017). When compulsory vaccination was introduced in Britain in the mid-19^th^ century, opponents claimed that people’s freedoms were being invaded by Parliament (Hussain et al., 2018). In mandatory medication switches from branded to generic medicines there are often backlash as patients fear side effects from the new brand and perceive their medicine options being removed due to a government cost-cutting strategy (Faasse et al., 2009). As a result, the nocebo effect frequently occurs in medicine brand changes (Weissenfeld et al., 2010).

## Health Scare Spread

The concerns about and nocebo reactions to a perceived health threat initially start as an individual response. However, these issues can grow and be spread to a wider population through traditional and social media. As will be discussed in the next sections, it is this spread and publicity that transforms a health incident to a health scare.

### Traditional Media

It has been claimed that the traditional media, such as newspapers and television, can turn a health incident into a crisis (Doeg, 1995). The media is central in the dissemination of health alarms (Burgess, 2008) and health scares are frequently characterised by mass media reporting creating panic about a health issue or intervention (Guillaume & Bath, 2004).

Observational studies clearly illustrate the impact of the media on spreading worry and adverse event reporting. Negative media coverage of the MMR vaccine by a local newspaper in the United Kingdom was associated with a decrease in vaccination rates by almost 14% in the area covered by the newspaper (Mason & Donnelly, 2000). Newspaper coverage of side effects was also found to be associated with an increase in adverse event reports from the HPV vaccination (Faasse et al., 2017). Media coverage discussing side effects from a generic antidepressant was associated with an increase in adverse event reporting, with television increasing the reporting rate by more than 210% compared to print media (MacKrill et al., 2019; MacKrill et al., 2020). Recently, the discussion of rare COVID-19 vaccine side effects in the media resulted in increased reporting of cardiac complaints, which were likely self-diagnosed (MacKrill, 2023). We identified two key factors responsible for the media’s ability to spread nocebo responding and anxiety about a health event: 1) the faming of the news item; and 2) the process of social modelling.

The way the media frames issues can influence the public’s expectations about a health event. The media is often people’s first source of information about a health threat and because it is considered to be a trusted source, the reporting has the ability to shape long-lasting expectations (Guillaume & Bath, 2004). However, it is usually negative expectations that are developed, as the media is more interested in stories about an intervention causing harm than stories about benefit (Kitzinger, 1999). Tobert and Newman (2016) give the example of how “Statins have very few adverse effects” is not newsworthy, but “Cholesterol drugs taken by millions are dangerous” often is. This media focus has resulted in strong expectations in the general public that statins are associated muscle pain and other side effects, resulting in high discontinuation rates (Matthews et al., 2016).

There is often an imbalance between how much media attention a health issue receives and its actual public health significance (Cooper & Roter, 2000). News articles about health threats disproportionately discuss toxic and environmental causes of illness, while neglecting lifestyle factors that are more common causes of illness (Frost et al., 1997). Even if experts or officials deny a link between a health event and an adverse reaction, the media have been known to report on an individual personal account of harm, allowing the perceived risk to enter public awareness (Kitzinger, 1998). Almost three quarters of British newspaper reports presented a mainly electromagnetic cause for complaints of symptoms from EMFs and used the experiences of particular people as examples (Eldridge-Thomas & Rubin, 2013).

Repeated reporting of a health issue can also be detrimental. The availability heuristic can influence people’s estimation of the probability of events due to how readily confirmatory examples can be brought to mind (Kahneman et al., 1982). When the media continuously highlights a health issue, examples of harm can be readily recalled causing people to overestimate its incidence (Gollust et al., 2019). Additionally, artificial balance can be created by the media. In the United Kingdom, the media often gave equal coverage to both sides of the MMR-autism debate, which led the public to assume there was equal evidence for each argument (Hargreaves et al., 2003).

News stories often simplify a health issue (Seale, 2003). In the case of genetically modified food, media coverage reduced the complexity of this issue into a simple conflict between organic versus processed foods. Organic food has been framed as safe, natural and nutritious, while the alternatives that are created through new technology are artificial, threatening and untrustworthy, which has been linked to a rising anti-genetic modification attitude in the general public (Lockie, 2006).

Media coverage is also able to spread adverse reaction reporting through the process of social modelling. It has been well documented in experimental placebo studies that seeing another person report side effects can influence the treatment outcome in the observer (Faasse & Petrie, 2013). Seeing a study confederate report side effects from a placebo tablet results in a reduced placebo effect as well as increased side effect reporting (Faasse et al., 2015). Similarly, after inhaling an inert substance described as a toxin, female participants reported more side effects if they saw a model also report side effects (Lorber et al., 2007; Mazzoni et al., 2010). Watching a model display more pain after a placebo cream was applied resulted in participants also reporting greater pain (Vögtle et al., 2013).

Media coverage replicates this social modelling effect on a larger scale. The act of seeing someone in a media story report medication side effects can lead to increased expectations in the observer that they too will experience this response (Faasse & Petrie, 2016). When participants were shown television coverage of people reporting negative health effects from wind turbine noise, they reported more symptoms and of higher intensity than those who watched a neutral information video (Crichton, Dodd, et al., 2014). This effect has also been found in research investigating EMFs (Bräscher et al., 2017; Witthöft & Rubin, 2013; Witthöft et al., 2018). This can also occur with written information. Participants who read a leaflet containing media warnings about environmental pollution and a case example of someone with Multiple Chemical Sensitivity, reported more side effects after inhaling an inert substance than those who did not receive prior warning (Winters et al., 2003).

### Social Media

More recently, social media has been a key medium in the spreading of negative beliefs about health interventions. Unlike traditional media, social media has allowed opponents of medical interventions to directly share their concerns, which are not required to factually accurate (Wilson & Keelan, 2013). For example, the vaccine-autism link has been shown to be discussed more frequently on social media than in online mainstream news sites (Jang et al., 2019). Similarly, in the 2014 Ebola crisis, news shared on the social media platform Reddit amplified panic and uncertainty surrounding Ebola, while traditional newspaper coverage was significantly less likely to produce panic-inducing coverage (Brown et al., 2019). In another study, mothers who do not support childhood vaccination were more likely to share opinions and negative information on social media compared to those who did support vaccination (McKeever et al., 2016).

Negative health information appears to spread more readily on social media than accurate or positive public health appeals. An analysis of news stories on Twitter found that false stories spread faster and more broadly than true stories, potentially due to them containing more novel information (Vosoughi et al., 2018). Exposure to misinformation on the internet about health threats can lead to negative expectations, which further reinforces opposition (Crichton & Petrie, 2015).

## Conclusion and Future Directions

This review provides the basis for how health scares are likely to develop and spread to wider populations. When a threatening incident occurs, background factors unique to an individual become more salient and influence expectations and health behaviours. Through examining health scare and nocebo effect literature, we identified specific influential background factors, namely conspiracy theories, trust in governmental agencies, anxiety, modern health worries, and a wariness of chemicals. Additionally, characteristics of incidents that appear to influence threat appraisals include being newly developed, not well understood or unseen threats, natural versus man-made, and out of personal control. Past research clearly shows that health concerns and adverse reactions can spread rapidly to a wider group of people through the attention of traditional and social media. This review has also provided a taxonomy to aid the grouping of health scares into common areas of concern. It is hoped that this taxonomy will help researchers differentiate between different types of health scares and encourage a greater analysis of how different factors way be involved in the development and resolution of specific scares.

It is evident that more research is needed on interventions to reduce the development and spread of health scares, in particular identifying the characteristics of media stories that intensify the levels of public concern and increase the likelihood a story will be shared widely. A cardinal characteristic of health scares is the perceived level of risk is disproportional to the actual level of risk. Unlike many areas of health where researchers wish to increase the public’s attention to risky behaviour or substances, in the case of health scares the need is to develop effective strategies to increase reassurance and alleviate public concern. It may be beneficial to provide an additional explanation of how beliefs and concerns can manifest symptoms and be misattributed to a treatment or other exposure, as this has been shown to be effective at reducing anxiety and symptom reporting (Crichton, Chapman, et al., 2014; MacKrill et al., 2021).

Social media has taken some steps to curb the spread of misinformation and scares by attaching a warning to posts that contain inaccurate information. In a similar vein, guidelines around the media’s reporting of side effects and potential health threats may be necessary in order to reduce the effect of social modelling and the spread of anxiety. There needs to be a balance between creating a newsworthy story but not needlessly exacerbating worries. Future research will further our understanding of the role of psychology in intensifying perceived health threats, which will aid the development of strategies to reduce the likelihood of health scares occurring in the future.

## References

[r1] Aaronovitch, D. (2010). *Voodoo histories: How conspiracy theory has shaped modern history*. Random House.

[r2] Ahmed, W., Vidal-Alaball, J., Downing, J., & Seguí, F. L. (2020). COVID-19 and the 5G conspiracy theory: Social network analysis of Twitter data. Journal of Medical Internet Research, 22(5), e19458. 10.2196/19458 32352383 PMC7205032

[r3] Armfield, J. M. (2007). When public action undermines public health: A critical examination of antifluoridationist literature. Australia and New Zealand Health Policy, 4(1), 25. 10.1186/1743-8462-4-25 18067684 PMC2222595

[r4] Banner, W. (2018). “Toxicohistrionics”: Flint, Michigan and the lead crisis. The Journal of Pediatrics, 197, 15–16. 10.1016/j.jpeds.2018.03.008 29656862

[r5] Barsky, A. J., Saintfort, R., Rogers, M. P., & Borus, J. F. (2002). Nonspecific medication side effects and the nocebo phenomenon. Journal of the American Medical Association, 287(5), 622–627. 10.1001/jama.287.5.622 11829702

[r6] Bearth, A., Cousin, M. E., & Siegrist, M. (2014). The consumer’s perception of artificial food additives: Influences on acceptance, risk and benefit perceptions. Food Quality and Preference, 38, 14–23. 10.1016/j.foodqual.2014.05.008

[r7] Bird, L. (2005). Bird flu scare. Nature Reviews Immunology, 5(1), 4. 10.1038/nri1539

[r8] Bluming, A. Z., & Tavris, C. (2009). Hormone replacement therapy: Real concerns and false alarms. Cancer Journal, 15(2), 93–104. 10.1097/PPO.0b013e31819e332a 19390302

[r9] Boone, N. W., Liu, L., Romberg-Camps, M. J., Duijsens, L., Houwen, C., van der Kuy, P. H. M., Janknegt, R., Peeters, R., Landewé, R. B. M., Winkens, B., & van Bodegraven, A. A. (2018). The nocebo effect challenges the non-medical infliximab switch in practice. European Journal of Clinical Pharmacology, 74(5), 655–661. 10.1007/s00228-018-2418-4 29368188 PMC5893662

[r10] Bräscher, A.-K., Raymaekers, K., Van den Bergh, O., & Witthöft, M. (2017). Are media reports able to cause somatic symptoms attributed to WiFi radiation? An experimental test of the negative expectation hypothesis. Environmental Research, 156, 265–271. 10.1016/j.envres.2017.03.040 28371755

[r11] Bräscher, A.-K., Schulz, S. M., Van den Bergh, O., & Witthöft, M. (2020). Prospective study of nocebo effects related to symptoms of idiopathic environmental intolerance attributed to electromagnetic fields (IEI-EMF). Environmental Research, 190, 110019. 10.1016/j.envres.2020.110019 32777274

[r12] Brown, D. K., Yoo, J., & Johnson, T. J. (2019). Spreading Ebola panic: Newspaper and social media coverage of the 2014 Ebola health crisis. Health Communication, 34(8), 811–817. 10.1080/10410236.2018.1437524 29474133

[r13] Burgess, A. (2004). *Cellular phones, public fears, and a culture of precaution*. Cambridge University Press.

[r14] Burgess, A. (2008). Health scares and risk awareness. In D. Wainwright (Ed.), *A sociology of health* (pp. 56-75). Sage.

[r15] Burgess, D. C., Burgess, M. A., & Leask, J. (2006). The MMR vaccination and autism controversy in United Kingdom 1998–2005: Inevitable community outrage or a failure of risk communication? Vaccine, 24(18), 3921–3928. 10.1016/j.vaccine.2006.02.033 16564116

[r16] Carstairs, C., & Elder, R. (2008). Expertise, health, and popular opinion: Debating water fluoridation, 1945–80. The Canadian Historical Review, 89(3), 345–371. 10.3138/chr.89.3.345

[r17] Chapman, S., St George, A., Waller, K., & Cakic, V. (2013). The pattern of complaints about Australian wind farms does not match the establishment and distribution of turbines: Support for the psychogenic ‘communicated disease’ hypothesis. PLoS One, 8, e76584. 10.1371/journal.pone.0076584 24146893 PMC3797792

[r18] Cooper, C. P., & Roter, D. L. (2000). “If it bleeds it leads”? Attributes of TV health news stories that drive viewer attention. Public Health Reports, 115(4), 331. 10.1093/phr/115.4.331 11059426 PMC1308573

[r19] Crichton, F., Chapman, S., Cundy, T., & Petrie, K. J. (2014). The link between health complaints and wind turbines: Support for the nocebo expectations hypothesis. Frontiers in Public Health, 2, 220. 10.3389/fpubh.2014.00220 25426482 PMC4227478

[r20] Crichton, F., Dodd, G., Schmid, G., Gamble, G., & Petrie, K. J. (2014). The power of positive and negative expectations to influence reported symptoms and mood during exposure to wind farm sound. Health Psychology, 33(12), 1588–1592. 10.1037/hea0000037 24274799

[r21] Crichton, F., & Petrie, K. J. (2015). Accentuate the positive: Counteracting psychogenic responses to media health messages in the age of the Internet. Journal of Psychosomatic Research, 79(3), 185–189. 10.1016/j.jpsychores.2015.04.014 25963037

[r22] David, A. S., & Wessely, S. C. (1995). The legend of Camelford: Medical consequences of a water pollution accident. Journal of Psychosomatic Research, 39(1), 1–9. 10.1016/0022-3999(94)00085-J 7760298

[r23] Devcich, D. A., Pedersen, I. K., & Petrie, K. J. (2007). You eat what you are: Modern health worries and the acceptance of natural and synthetic additives in functional foods. Appetite, 48(3), 333–337. 10.1016/j.appet.2006.09.014 17137678

[r24] Dickson-Spillmann, M., Siegrist, M., & Keller, C. (2011). Attitudes toward chemicals are associated with preference for natural food. Food Quality and Preference, 22(1), 149–156. 10.1016/j.foodqual.2010.09.001

[r25] Dodes, J. E. (2001). The amalgam controversy: An evidence-based analysis. The Journal of the American Dental Association, 132(3), 348–356. 10.14219/jada.archive.2001.0178 11258092

[r26] Doeg, C. (1995). *Crisis management in the food and drinks industry*. Chapman & Hall.

[r27] Drake, F. (2006). Mobile phone masts: Protesting the scientific evidence. Public Understanding of Science, 15(4), 387–410. 10.1177/0963662506057246

[r28] Eldridge-Thomas, B., & Rubin, G. J. (2013). Idiopathic environmental intolerance attributed to electromagnetic fields: A content analysis of British newspaper reports. PLoS One, 8(6), e65713. 10.1371/journal.pone.0065713 23799038 PMC3683033

[r29] Elwood, M., & Wood, A. W. (2019). Health effects of radiofrequency electromagnetic energy. The New Zealand Medical Journal, 132(1501), 64–72. 31465329

[r30] Engel, C. C., Locke, S., Reissman, D. B., DeMartino, R., Kutz, I., McDonald, M., & Barsky, A. J. (2007). Terrorism, trauma, and mass casualty triage: How might we solve the latest mind-body problem? Biosecurity and Bioterrorism, 5(2), 155–163. 10.1089/bsp.2007.0004 17608601

[r31] Entine, J. (2011). *Scared to death: How Chemophobia threatens public health.* American Council on Science and Health.

[r32] Faasse, K., Cundy, T., & Petrie, K. J. (2009). Thyroxine: Anatomy of a health scare. BMJ, 339, b5613. 10.1136/bmj.b5613 20040484

[r33] Faasse, K., Gamble, G., Cundy, T., & Petrie, K. J. (2012). Impact of television coverage on the number and type of symptoms reported during a health scare: A retrospective pre–post observational study. BMJ Open, 2(4), e001607. 10.1136/bmjopen-2012-001607 22904334 PMC3425900

[r34] Faasse, K., Grey, A., Jordan, R., Garland, S., & Petrie, K. J. (2015). Seeing is believing: Impact of social modeling on placebo and nocebo responding. Health Psychology, 34(8), 880–885. 10.1037/hea0000199 25545042

[r35] Faasse, K., Martin, L. R., Grey, A., Gamble, G., & Petrie, K. J. (2016). Impact of brand or generic labeling on medication effectiveness and side effects. Health Psychology, 35(2), 187–190. 10.1037/hea0000282 26462056

[r36] Faasse, K., & Petrie, K. J. (2013). The nocebo effect: Patient expectations and medication side effects. Postgraduate Medical Journal, 89(1055), 540–546. 10.1136/postgradmedj-2012-131730 23842213

[r37] Faasse, K., & Petrie, K. J. (2016). From me to you: The effect of social modeling on treatment outcomes. Current Directions in Psychological Science, 25(6), 438–443. 10.1177/0963721416657316

[r38] Faasse, K., Porsius, J. T., Faasse, J., & Martin, L. R. (2017). Bad news: The influence of news coverage and Google searches on Gardasil adverse event reporting. Vaccine, 35(49), 6872–6878. 10.1016/j.vaccine.2017.10.004 29128382

[r39] Flanders, R. A. (1992). Mercury in dental amalgam – A public health concern? Journal of Public Health Dentistry, 52(5), 303–311. 10.1111/j.1752-7325.1992.tb02293.x 1404077

[r40] Foster, K. R. (2019, September 16). 5G Is coming: How worried should we be about the health risks? *Scientific American**.* https://blogs.scientificamerican.com/observations/5g-is-coming-how-worried-should-we-be-about-the-health-risks/

[r41] Frewer, L. J., Miles, S., & Marsh, R. (2002). The media and genetically modified foods: Evidence in support of social amplification of risk. Risk Analysis, 22(4), 701–711. 10.1111/0272-4332.00062 12224744

[r42] Frost, K., Frank, E., & Maibach, E. (1997). Relative risk in the news media: A quantification of misrepresentation. American Journal of Public Health, 87(5), 842–845. 10.2105/AJPH.87.5.842 9184517 PMC1381061

[r43] Furnham, A. (2007). Are modern health worries, personality and attitudes to science associated with the use of complementary and alternative medicine? British Journal of Health Psychology, 12(2), 229–243. 10.1348/135910706X100593 17456283

[r44] Gollust, S. E., Fowler, E. F., & Niederdeppe, J. (2019). Television news coverage of public health issues and implications for public health policy and practice. Annual Review of Public Health, 40, 167–185. 10.1146/annurev-publhealth-040218-044017 30633711

[r45] Guillaume, L. R., & Bath, P. A. (2004). The impact of health scares on parents’ information needs and preferred information sources: A case study of the MMR vaccine scare. Health Informatics Journal, 10(1), 5–22. 10.1177/1460458204040664

[r46] Haas, J. S., Miglioretti, D. L., Geller, B., Buist, D. S., Nelson, D. E., Kerlikowske, K., Carney, P. A., Dash, S., Breslau, E. S., & Ballard-Barbash, R. (2007). Average household exposure to newspaper coverage about the harmful effects of hormone therapy and population-based declines in hormone therapy use. Journal of General Internal Medicine, 22(1), 68–73. 10.1007/s11606-007-0122-7 17351842 PMC1824785

[r47] Haen, D. (2014). The paradox of E-numbers: Ethical, aesthetic, and cultural concerns in the Dutch discourse on food additives. Journal of Agricultural & Environmental Ethics, 27(1), 27–42. 10.1007/s10806-013-9440-4

[r48] Hargreaves, I. L., Lewis, J., & Speers, T. (2003). *Towards a better map: Science, the public and the media.* Economic and Social Research Council.

[r49] Hooker, C. (2008). SARS as a health scare. In H. Ali & R. Keil (Eds.), *Networked disease: Emerging infectious disease in the global city* (pp. 123-137). Wiley-Blackwell.

[r50] Hooker, C. (2010). Health scares: Professional priorities. Health, 14(1), 3–21. 10.1177/1363459309341875 20051427

[r51] Howat, P., Binns, C., & Jancey, J. (2015). New international review supports community water fluoridation as an effective and safe dental health promotion measure. Health Promotion Journal of Australia, 26(1), 1–3. 10.1071/HEv26n1_ED 26149252

[r52] Hussain, A., Ali, S., Ahmed, M., & Hussain, S. (2018). The anti-vaccination movement: A regression in modern medicine. Cureus, 10(7), e2919. 10.7759/cureus.2919 30186724 PMC6122668

[r53] INTERPHONE Study Group. (2010). Brain tumour risk in relation to mobile telephone use: Results of the INTERPHONE international case–control study. International Journal of Epidemiology, 39(3), 675–694. 10.1093/ije/dyq079 20483835

[r54] Jacob, C. J., Lok, C., Morley, K., & Powell, D. A. (2011). Government management of two media-facilitated crises involving dioxin contamination of food. Public Understanding of Science, 20(2), 261–269. 10.1177/0963662509355737 21657135

[r55] Jang, S. M., Mckeever, B. W., Mckeever, R., & Kim, J. K. (2019). From social media to mainstream news: The information flow of the vaccine-autism controversy in the US, Canada, and the UK. Health Communication, 34(1), 110–117. 10.1080/10410236.2017.1384433 29028371

[r56] Jolley, D., & Douglas, K. M. (2014). The effects of anti-vaccine conspiracy theories on vaccination intentions. PLoS One, 9(2), e89177. 10.1371/journal.pone.0089177 24586574 PMC3930676

[r57] Kahneman, D., Slovic, P., & Tversky, A. (Eds.). (1982). *Judgment under uncertainty: Heuristics and biases*. Cambridge University Press. 10.1126/science.185.4157.1124 17835457

[r58] Kinman, G., & Griffin, M. (2008). Psychosocial factors and gender as predictors of symptoms associated with sick building syndrome. Stress and Health, 24(2), 165–171. 10.1002/smi.1175

[r59] Kitzinger, J. (1998). Silenced voices and false memories. In C. Carter, G. Branston, & S. Allan (Eds.), *News, gender, and power* (pp. 186-204). Routledge.

[r60] Kitzinger, J. (1999). Researching risk and the media. Health Risk & Society, 1(1), 55–69. 10.1080/13698579908407007

[r61] Klemm, C., Das, E., & Hartmann, T. (2016). Swine flu and hype: A systematic review of media dramatization of the H1N1 influenza pandemic. Journal of Risk Research, 19(1), 1–20. 10.1080/13669877.2014.923029

[r62] Leask, A., Delpech, V., & McAnulty, J. (2003). Anthrax and other suspect powders: Initial responses to an outbreak of hoaxes and scares. New South Wales Public Health Bulletin, 14(12), 218–221. 10.1071/NB03059 14981556

[r63] Lockie, S. (2006). Capturing the sustainability agenda: Organic foods and media discourses on food scares, environment, genetic engineering, and health. Agriculture and Human Values, 23(3), 313–323. 10.1007/s10460-006-9007-3

[r64] Lorber, W., Mazzoni, G., & Kirsch, I. (2007). Illness by suggestion: Expectancy, modeling, and gender in the production of psychosomatic symptoms. Annals of Behavioral Medicine, 33(1), 112–116. 10.1207/s15324796abm3301_13 17291177

[r65] MacKrill, K. (2021). *Improving the understanding and measurement of the nocebo effect* [Doctoral thesis, The University of Auckland]. ResearchSpace@Auckland. https://hdl.handle.net/2292/57853

[r66] MacKrill, K. (2023). Impact of media coverage on side effect reports from the COVID-19 vaccine. Journal of Psychosomatic Research, 164, 111093. 10.1016/j.jpsychores.2022.111093 36435094 PMC9670676

[r67] MacKrill, K., Gamble, G. D., Bean, D. J., Cundy, T., & Petrie, K. J. (2019). Evidence of a media-induced nocebo response following a nationwide antidepressant drug switch. Clinical Psychology in Europe, 1(1), e29642. 10.32872/cpe.v1i1.29642

[r68] MacKrill, K., Gamble, G. D., & Petrie, K. (2020). The effect of television and print news stories on the nocebo responding following a generic medication switch. Clinical Psychology in Europe, 2(2), e2623. 10.32872/cpe.v2i2.2623 36397827 PMC9645488

[r69] MacKrill, K., Morrison, Z., & Petrie, K. J. (2021). Increasing and dampening the nocebo response following medicine-taking: A randomised controlled trial. Journal of Psychosomatic Research, 150, 110630. 10.1016/j.jpsychores.2021.110630 34607238

[r70] MacKrill, K., & Petrie, K. J. (2018). What is associated with increased side effects and lower perceived efficacy following switching to a generic medicine? A New Zealand cross-sectional patient survey. BMJ Open, 8(10), e023667. 10.1136/bmjopen-2018-023667 30341138 PMC6196872

[r71] Martin, L. R., & Petrie, K. J. (2017). Understanding the dimensions of anti-vaccination attitudes: The Vaccination Attitudes Examination (VAX) Scale. Annals of Behavioral Medicine, 51(5), 652–660. 10.1007/s12160-017-9888-y 28255934

[r72] Mason, B. W., & Donnelly, P. D. (2000). Impact of a local newspaper campaign on the uptake of the measles mumps and rubella vaccine. Journal of Epidemiology and Community Health, 54(6), 473–474. 10.1136/jech.54.6.473 10818125 PMC1731691

[r73] Matthews, A., Herrett, E., Gasparrini, A., Van Staa, T., Goldacre, B., Smeeth, L., & Bhaskaran, K. (2016). Impact of statin related media coverage on use of statins: Interrupted time series analysis with UK primary care data. BMJ, 353, i3283. 10.1136/bmj.i3283 27353418 PMC4925917

[r74] Mazzoni, G., Foan, L., Hyland, M. E., & Kirsch, I. (2010). The effects of observation and gender on psychogenic symptoms. Health Psychology, 29(2), 181–185. 10.1037/a0017860 20230091

[r75] McKeever, B. W., McKeever, R., Holton, A. E., & Li, J.-Y. (2016). Silent majority: Childhood vaccinations and antecedents to communicative action. Mass Communication and Society, 19(4), 476–498. 10.1080/15205436.2016.1148172

[r76] McMahan, S., & Meyer, J. A. (1995). Symptom prevalence and worry about high voltage transmission lines. Environmental Research, 70(2), 114–118. 10.1006/enrs.1995.1055 8674479

[r77] Mendelson, M. B., Catano, V. M., & Kelloway, K. (2000). The role of stress and social support in Sick Building Syndrome. Work and Stress, 14(2), 137–155. 10.1080/026783700750051658

[r78] Molin, C. (1992). Amalgam – Fact and fiction. European Journal of Oral Sciences, 100(1), 66–73. 10.1111/j.1600-0722.1992.tb01811.x 1557606

[r79] Oliver, J. E., & Wood, T. (2014). Medical conspiracy theories and health behaviors in the United States. JAMA Internal Medicine, 174(5), 817–818. 10.1001/jamainternmed.2014.190 24638266

[r80] Ooi, P. L., & Goh, K. T. (1997). Sick building syndrome: An emerging stress-related disorder? International Journal of Epidemiology, 26(6), 1243–1249. 10.1093/ije/26.6.1243 9447404

[r81] Owens, K., & Feldman, J. (2004). Getting the drift on chemical trespass. Pesticides and You, 24(2), 16–21.

[r82] Paarlberg, R. (2021). *Resetting the table: Straight talk about the food we grow and eat*. Knopf.

[r83] Page, L. A., Petrie, K. J., & Wessely, S. C. (2006). Psychosocial responses to environmental incidents: A review and proposed typology. Journal of Psychosomatic Research, 60(4), 413–422. 10.1016/j.jpsychores.2005.11.008 16581367

[r84] Paytubi, S., Benavente, Y., Montoliu, A., Binefa, G., Brotons, M., Ibanez, R., Ochoa, C., Peremiquel-Trillas, P., Serrano, B., Travier, N., Alemany, L., & Costas, L. (2022). Everything causes cancer? Beliefs and attitudes towards cancer prevention among anti-vaxxers, flat earthers, and reptilian conspiracists: Online cross sectional survey. BMJ, 379, e072561. 10.1136/bmj-2022-072561 36543351 PMC9768817

[r85] Petrie, K. J., Broadbent, E. A., Kley, N., Moss-Morris, R., Horne, R., & Rief, W. (2005). Worries about modernity predict symptom complaints after environmental pesticide spraying. Psychosomatic Medicine, 67(5), 778–782. 10.1097/01.psy.0000181277.48575.a4 16204438

[r86] Petrie, K. J., Moss-Morris, R., Grey, C., & Shaw, M. (2004). The relationship of negative affect and perceived sensitivity to symptom reporting following vaccination. British Journal of Health Psychology, 9(1), 101–111. 10.1348/135910704322778759 15006204

[r87] Petrie, K. J., & Rief, W. (2019). Psychobiological mechanisms of placebo and nocebo effects: Pathways to improve treatments and reduce side effects. Annual Review of Psychology, 70(1), 599–625. 10.1146/annurev-psych-010418-102907 30110575

[r88] Petrie, K. J., Sivertsen, B., Hysing, M., Broadbent, E., Moss-Morris, R., Eriksen, H. R., & Ursin, H. (2001). Thoroughly modern worries: The relationship of worries about modernity to reported symptoms, health and medical care utilization. Journal of Psychosomatic Research, 51(1), 395–401. 10.1016/S0022-3999(01)00219-7 11448708

[r89] Petrie, K. J., & Wessely, S. (2002). Modern worries, new technology, and medicine. BMJ, 324, 690. 10.1136/bmj.324.7339.690 11909772 PMC1122629

[r90] Petrie, K. J., & Wessely, S. (2004). Getting well from water. BMJ, 329, 1417. 10.1136/bmj.329.7480.1417 15604162 PMC535954

[r91] Petts, J., & Niemeyer, S. (2004). Health risk communication and amplification: Learning from the MMR vaccination controversy. Health Risk & Society, 6(1), 7–23. 10.1080/13698570410001678284

[r92] Reekie, D. (2017). Fear of fluoride. British Dental Journal, 222(1), 16–18. 10.1038/sj.bdj.2017.23 28084346 PMC7091724

[r93] Rief, W., Glaesmer, H., Baehr, V., Broadbent, E., Brähler, E., & Petrie, K. J. (2012). The relationship of modern health worries to depression, symptom reporting and quality of life in a general population survey. Journal of Psychosomatic Research, 72(4), 318–320. 10.1016/j.jpsychores.2011.11.017 22405228

[r94] Romer, D., & Jamieson, J. H. (2020). Conspiracy theories as a barrier to controlling the spread of COVID-19 in the U.S. Social Science & Medicine, 263, 113356. 10.1016/j.socscimed.2020.113356 32967786 PMC7502362

[r128] Roy, S., Petrie, K. J., Gamble, G., & Edwards, M. A. (2023). Did a nocebo effect contribute to the rise in special education enrollment following the Flint, Michigan Water Crisis? Clinical Psychology in Europe, 5(1), e9577. 10.32872/cpe.9577 37065004 PMC10103158

[r95] Rubin, G. J., & Dickmann, P. (2010). How to reduce the impact of “low-risk patients” following a bioterrorist incident: Lessons from SARS, anthrax, and pneumonic plague. Biosecurity and Bioterrorism, 8(1), 37–43. 10.1089/bsp.2009.0059 20230231

[r96] Rubin, G. J., Nieto‐Hernandez, R., & Wessely, S. (2010). Idiopathic environmental intolerance attributed to electromagnetic fields (formerly ‘electromagnetic hypersensitivity’): An updated systematic review of provocation studies. Bioelectromagnetics, 31(1), 1–11. 10.1002/bem.20536 19681059

[r97] Rubin, G. J., Page, L., Morgan, O., Pinder, R. J., Riley, P., Hatch, S., Maguire, H., Catchpole, M., Simpson, J., & Wessely, S. (2007). Public information needs after the poisoning of Alexander Litvinenko with polonium-210 in London: Cross sectional telephone survey and qualitative analysis. BMJ, 335(7630), 1143. 10.1136/bmj.39367.455243.BE 17975252 PMC2099556

[r98] Rubin, G. J., Webster, R., Amlot, R., Carter, H., Weston, D., & Wessely, S. (2020). Public responses to the Salisbury Novichok incident: A cross-sectional survey of anxiety, anger, uncertainty, perceived risk and avoidance behaviour in the local community. BMJ Open, 10(9), e036071. 10.1136/bmjopen-2019-036071 32978184 PMC7520835

[r99] Saleh, R., Bearth, A., & Siegrist, M. (2019). “Chemophobia” today: Consumers’ knowledge and perceptions of chemicals. Risk Analysis, 39(12), 2668–2682. 10.1111/risa.13375 31290192

[r100] Sanders, L. (2020, May 27). The difference between what Republicans and Democrats believe to be true about COVID-19. *YouGov**.* https://today.yougov.com/topics/politics/articles-reports/2020/05/26/republicans-democrats-misinformation

[r101] Scherer, L. D., McPhetres, J., Pennycook, G., Kempe, A., Allen, L. A., Knoepke, C. E., Tate, C. E., & Matlock, D. D. (2021). Who is susceptible to online health misinformation? A test of four psychosocial hypotheses. Health Psychology, 40(4), 274–284. 10.1037/hea0000978 33646806

[r102] Seale, C. (2003). Health and media: An overview. Sociology of Health & Illness, 25(6), 513–531. 10.1111/1467-9566.t01-1-00356 12919443

[r103] Shaw, A. (2002). “It just goes against the grain.” Public understandings of genetically modified (GM) food in the UK. Public Understanding of Science, 11(3), 273–291. 10.1088/0963-6625/11/3/305 12430532

[r104] Siegrist, M., & Bearth, A. (2019). Chemophobia in Europe and reasons for biased risk perceptions. Nature Chemistry, 11(12), 1071–1072. 10.1038/s41557-019-0377-8 31700123

[r105] Slovic, P. (1987). Perception of risk. Science, 236(4799), 280–285. 10.1126/science.3563507 3563507

[r106] Southwell, B. G., Niederdeppe, J., Cappella, J. N., Gaysynsky, A., Kelley, D. E., Oh, A., Peterson, E. B., & Chou, W. S. (2019). Misinformation as a misunderstood challenge to public health. American Journal of Preventive Medicine, 57(2), 282–285. 10.1016/j.amepre.2019.03.009 31248741

[r107] Swerdlow, A. J., Feychting, M., Green, A. C., Kheifets, L., Savitz, D. A., & International Commission for Non-Ionizing Radiation Protection Standing Committee on Epidemiology. (2011). Mobile phones, brain tumors, and the interphone study: Where are we now? Environmental Health Perspectives, 119(11), 1534–1538. 10.1289/ehp.1103693 22171384 PMC3226506

[r108] Tausczik, Y., Faasse, K., Pennebaker, J. W., & Petrie, K. J. (2012). Changes in anxiety and information seeking following the H1N1 outbreak: An analysis of web blogs and Wikipedia Searches. Journal of Health Communication, 27(2), 179–185. 10.1080/10410236.2011.571759 21827326

[r109] Tobert, J. A., & Newman, C. B. (2016). The nocebo effect in the context of statin intolerance. Journal of Clinical Lipidology, 10(4), 739–747. 10.1016/j.jacl.2016.05.002 27578103

[r110] United States Food and Drug Administration. (2020). *Do cell phones pose a health hazard**?* https://www.fda.gov/radiation-emitting-products/cell-phones/do-cell-phones-pose-health-hazard

[r111] Uscinski, J. E., Klofstad, C., & Atkinson, M. D. (2016). What drives conspiratorial beliefs? The role of informational cues and predispositions. Political Research Quarterly, 69(1), 57–71. 10.1177/1065912915621621

[r112] van Prooijen, J.-W., & Douglas, K. M. (2017). Conspiracy theories as part of history: The role of societal crisis situations. Memory Studies, 10(3), 323–333. 10.1177/1750698017701615 29081831 PMC5646574

[r113] Vernia, P., Di Camillo, M., Foglietta, T., Avallone, V. E., & De Carolis, A. (2010). Diagnosis of lactose intolerance and the “nocebo” effect: The role of negative expectations. Digestive and Liver Disease, 42(9), 616–619. 10.1016/j.dld.2010.02.005 20227928

[r114] Vögtle, E., Barke, A., & Kröner-Herwig, B. (2013). Nocebo hyperalgesia induced by social observational learning. Pain, 154(8), 1427–1433. 10.1016/j.pain.2013.04.041 23707275

[r115] Vosoughi, S., Roy, D., & Aral, S. (2018). The spread of true and false news online. Science, 359(6380), 1146–1151. 10.1126/science.aap9559 29590045

[r116] Watson, D., & Pennebaker, J. W. (1989). Health complaints, stress and distress: Exploring the central role of negative affectivity. Psychological Review, 96(2), 234–254. 10.1037/0033-295X.96.2.234 2710874

[r117] Webster, R. K., Weinman, J., & Rubin, G. J. (2018). Medicine‐related beliefs predict attribution of symptoms to a sham medicine: A prospective study. British Journal of Health Psychology, 23(2), 436–454. 10.1111/bjhp.12298 29405507 PMC5900880

[r118] Weissenfeld, J., Stock, S., Lüngen, M., & Gerber, A. (2010). The nocebo effect: A reason for patients’ non-adherence to generic substitution? *Die Pharmazie – An International*. Journal of Pharmaceutical Sciences, 65(7), 451–456. 10.1691/ph.2010.9749 20662309

[r119] Whitworth, E., Druckman, A., & Woodward, A. (2017). Food scares: A comprehensive categorisation. British Food Journal, 119(1), 131–142. 10.1108/BFJ-06-2016-0263

[r120] Whorton, J. C. (1978). The hygiene of the wheel: An episode in Victorian sanitary science. Bulletin of the History of Medicine, 52(1), 61–88. 352453

[r121] Wills, B., Leikin, J. B., Rhee, J., Tameling, C., & Saeedi, B. (2008). Analysis of suspicious powders following the post 9/11 anthrax scare. Journal of Medical Toxicology, 4(2), 93–95. 10.1007/BF03160961 18570168 PMC3550131

[r122] Wilson, K., & Keelan, J. (2013). Social media and the empowering of opponents of medical technologies: The case of anti-vaccinationism. Journal of Medical Internet Research, 15(5), e103. 10.2196/jmir.2409 23715762 PMC3668617

[r123] Winters, W., Devriese, S., Van Diest, I., Nemery, B., Veulemans, H., Eelen, P., Van de Woestijne, K., & Van den Bergh, O. (2003). Media warnings about environmental pollution facilitate the acquisition of symptoms in response to chemical substances. Psychosomatic Medicine, 65(3), 332–338. 10.1097/01.PSY.0000041468.75064.BE 12764204

[r124] Witthöft, M., Freitag, I., Nußbaum, C., Bräscher, A.-K., Jasper, F., Bailer, J., & Rubin, G. J. (2018). On the origin of worries about modern health hazards: Experimental evidence for a conjoint influence of media reports and personality traits. Psychology & Health, 33(3), 361–380. 10.1080/08870446.2017.1357814 28758796

[r125] Witthöft, M., & Rubin, G. J. (2013). Are media warnings about the adverse health effects of modern life self-fulfilling? An experimental study on idiopathic environmental intolerance attributed to electromagnetic fields (IEI-EMF). Journal of Psychosomatic Research, 74(3), 206–212. 10.1016/j.jpsychores.2012.12.002 23438710

[r126] World Health Organisation. (2014). *Electromagnetic fields and public health: Mobile phones*. https://www.who.int/news-room/fact-sheets/detail/electromagnetic-fields-and-public-health-mobile-phones

[r127] World Health Organisation. (2023, January 6). *Avian Influenza Weekly Update Number 877**.* https://iris.who.int/bitstream/handle/10665/365675/AI-20230106.pdf

